# Simultaneously maximizing root/mycorrhizal growth and phosphorus uptake by cotton plants by optimizing water and phosphorus management

**DOI:** 10.1186/s12870-018-1550-8

**Published:** 2018-12-05

**Authors:** Wenxuan Mai, Xiangrong Xue, Gu Feng, Changyan Tian

**Affiliations:** 1 Xinjiang Institute of Ecology and Geography, Chinese Academy of Sciences, Urumqi, 830011 China; 2 State Key Laboratory of Oasis Ecology and Desert Environment, Urumqi, 830011 China; 3 College of Resources and Environment, China Agricultural University, Beijing, 100083 China; 4 Changji National Agricultural Science and Technology Park, Changji, 831100 China

**Keywords:** P uptake, Root, Hyphal density, Cotton

## Abstract

**Background:**

There are two plant phosphorus (P)-uptake pathways, namely the direct P uptake by roots and the indirect P uptake through arbuscular mycorrhizal fungi (AMF). Maximizing the efficiency of root and AMF processes associated with P acquisition by adjusting soil conditions is important for simultaneously ensuring high yields and the efficient use of available P.

**Results:**

A root box experiment was conducted in 2015 and 2016. The aim was to investigate the effects of different P and soil water conditions on root/mycorrhizal growth and P uptake by cotton plants. Hyphal growth was induced in well-watered soil, but decreased with increasing P concentrations. Additionally, P fertilizers regulated root length only under well-watered conditions, with the longest roots observed in response to 0.2 g P_2_O_5_ kg^− 1^. In contrast, root elongation was essentially unaffected by P fertilizers under drought conditions. And soil water in general had more significant effects on root and hyphal growth than phosphorus levels. In well-watered soil, the application of P significantly increased the cotton plant P uptake, but there were no differences between the effects of 0.2 and 1 g P_2_O_5_ kg^− 1^. So optimizing phosphorus inputs and soil water can increase cotton growth and phosphorus uptake by maximizing the efficiency of phosphorus acquisition by roots/mycorrhizae.

**Conclusions:**

Soil water and P contents of 19–24% and 20–25 mg kg^− 1^, respectively, simultaneously maximized root/mycorrhizal growth and P uptake by cotton plants.

**Electronic supplementary material:**

The online version of this article (10.1186/s12870-018-1550-8) contains supplementary material, which is available to authorized users.

## Background

High inputs and outputs and low nutrient use efficiency are typical characteristics of intensive farming systems in China [1]. For example, to overcome the effects of phosphorus (P)-deficient soils and obtain high crop yields, a large amount of P fertilizer has been applied to farmlands over the last 20 years, which has resulted in farmland soils having an average P content exceeding 242 kg ha^− 1^ [2]. However, the efficiency of P fertilizer use has decreased from 15 to 20% in the 1990s to 11.6% in 2003 [3]. Considering that P fertilizers represent a non-renewable resource, improving the efficiency of P use is of vital importance for ensuring sustainable agricultural production.

Most plants consist of two P-uptake pathways, namely the direct root P-uptake pathway and the arbuscular mycorrhizalfungi (AMF) P-uptake pathway [4, 5]. Most P fertilizers are immobilized in soils because P is strongly adsorbed to iron and aluminum cations at low soil pH [6, 7] and to calcium at high soil pH [8]. This is also the key reason for the low efficiency of P fertilizer use [9–11]. Thus, root architectural features and the growth of mycorrhizal hyphae are important for maximizing the acquisition of P because the root and mycorrhizal systems with a relatively high surface area are able to effectively use a given volume of soil [12].

Liao et al. [13–15] completed a series of experiments to prove there is a close relationship between bean root architecture and tolerance to low soil P levels. The relatively shallow bean root system is conducive for obtaining P, and provides evidence for the ideal root architecture model. On intensively farmed land, it is unclear whether the AMF P-uptake pathway contributes significantly to crop production. A key reason for this uncertainty is the fact mycorrhizal colonization decreases as the soil P content increases [16]. However, a series of field studies revealed that many AMF are present in high-yielding farmland soil. Moreover, the AMF are associated with a relatively high colonization rate and considerably affect crop P acquisition from the soil [17]. An investigation involving the ^32^P isotope confirmed the AMF P-uptake pathway may provide > 20% of the P obtained by maize plants, even under conditions of high P content (i.e., > 50 mg kg^− 1^ according to the Olsen-P method) [18].

Plant root growth is influenced by soil P and water contents [19]. In many plant species, P deficiency decreases primary root growth and increases the length and density of root hairs and lateral roots [20, 21] to increase the root–soil contact, which will enhance P uptake and the use of the available soil volume [8, 22]. The irrigation of crop plants induces significant changes in the growth and distribution of root systems, with important consequences for both nutrient uptake and crop growth. We previously reported that 49% of the cotton root length is distributed within 10 cm of the soil surface under drip irrigation conditions, while under flood irrigation conditions, this proportion is only 31% [23]. There is still some controversy regarding the effects of soil water conditions on AMF [24]. Although most studies have concluded that drought stress can promote the growth of mycorrhizal fungi [25, 26], at least one investigation produced contradictory results [27]. Additionally, other studies have indicated that mycorrhizae are unaffected by water conditions, but are influenced by available P contents in soils [28, 29]. Therefore, the response of AMF to available soil water is complex, with varying results obtained under diverse experimental conditions. Further research will be needed to clarify these responses.

With gradually decreasing availability of water resources, water-conserving irrigation methods, especially drip irrigation, have been widely promoted for crop production in China. For example, in Xinjiang, which represents a typical arid irrigation area, drip irrigation is used on > 60% of the cropland, and the proportion continues to increase. However, there has been no change in the method used to apply P fertilizers (i.e., as a base fertilizer), which have accumulated in the soil as in other places in China over the past 30 years. Consequently, maximizing the efficiency of the direct root and AMF P-uptake pathways and optimizing P nutrient input and soil water conditions are critical for ensuring sustainable high-yielding cotton production. Moreover, because drip irrigation enables the precise management of soil water content, it represents a unique option for improving the efficiency of P fertilizer use via its effects on root morphology. We hypothesized that the cotton phosphorus uptake can be increased through increasing the growth of cotton roots and mycorrhizal simultaneously by optimizing water and phosphorus management.

The objective of the present study was to determine the effects of P fertilizers and soil water conditions on the spatial distribution of cotton roots, mycorrhizal fungi growth, and P uptake by cotton plants. We also aimed to determine the optimal soil P and water contents for maximizing P uptake by cotton plants via root–mycorrhizae interactions.

## Results

### Root length and hyphal density

**Fig. 1:**
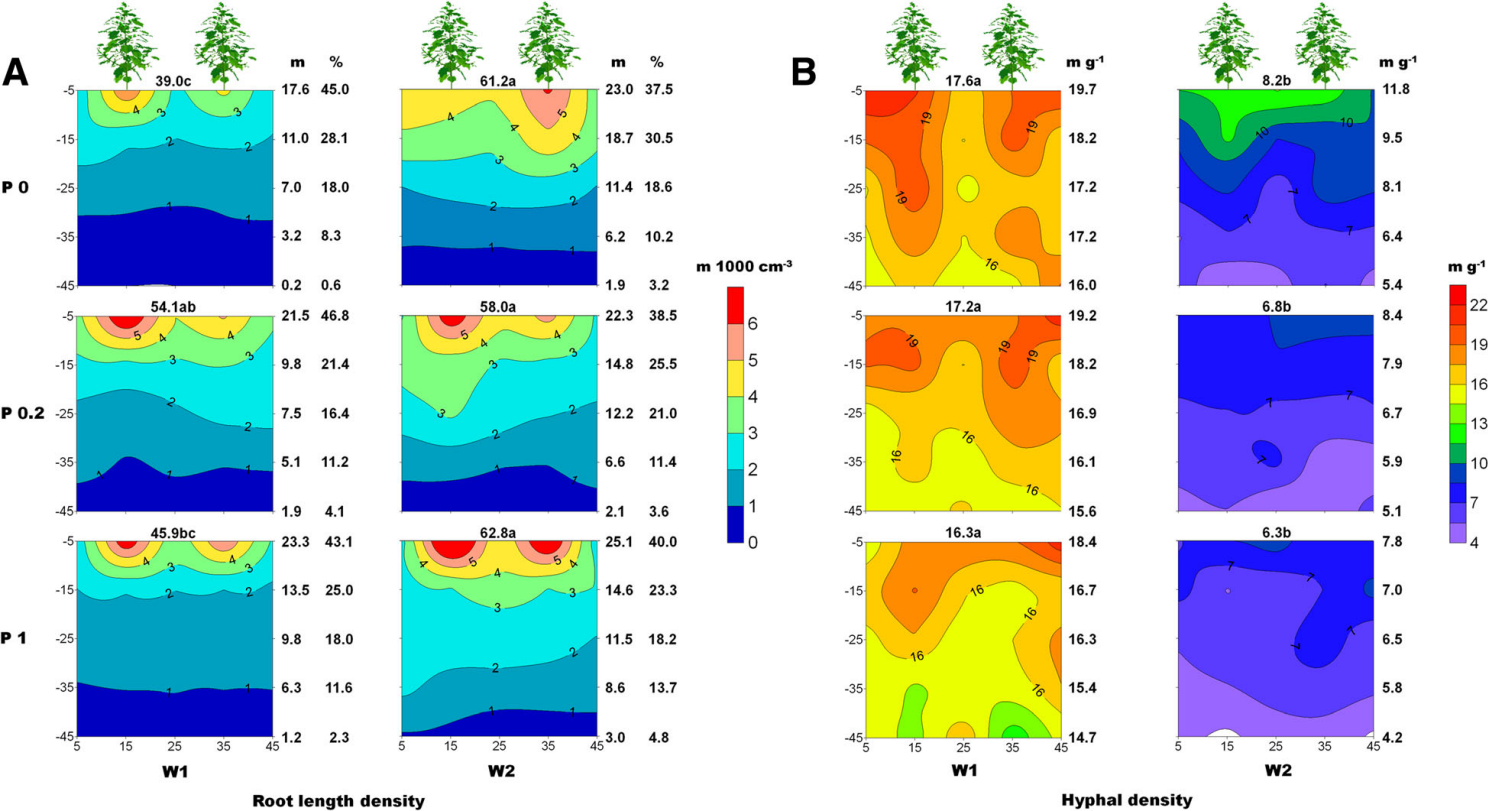
Effects of soil water and phosphorus contents on the spatial distributions of cotton root length (**a**) and hyphal density (**b**). Data at the top of each diagram correspond to the total cotton root length (**a**) and mean hyphal density (**b**) in the root boxes. Different letters indicate significant differences at the 0.05 level among different treatments. **a** Data on the right side of each diagram represent the cotton root length (m) in each soil layer (10 cm layer) and the ratio (%) to the total root length. **b** Data on the right side of each diagram correspond to the average hyphal density (m g^− 1^) in different soil layers. Data are presented as the mean values over 2 years (2015 and 2016) (same as in the other figures)

Cotton roots were longer and grew more deeply into the soil profile under water-limited conditions (Fig. 1a). Moreover, the effect of the P fertilizer on root elongation depended on the soil water conditions. In well-watered soil, cotton roots were longest (54.1 m box^− 1^) under the P0.2 treatment. In contrast, root length was almost unaffected by P content under drought conditions.

In all soil layers, the hyphal density under well-watered conditions was higher than that under drought conditions. Additionally, hyphal density decreased with increasing P content, although the changes were smaller than those induced by different water levels (Fig. 1b). For example, in the 0–10 cm soil layer, under well-watered conditions, the hyphal densities were 19.7, 19.2, and 18.4 m g^− 1^, while under drought conditions they were 11.8, 8.4, 7.8 m g^− 1^ in response to the P0, P0.2, and P1 treatments, respectively. The differences in the hyphal densities between the well-watered and drought conditions following the P0, P0.2, and P1 treatments (i.e., 40.1, 52.3, and 57.6% lower, respectively) were significant. Moreover, hyphal density decreased at increasing soil depths under different treatment conditions.

**Fig. 2:**
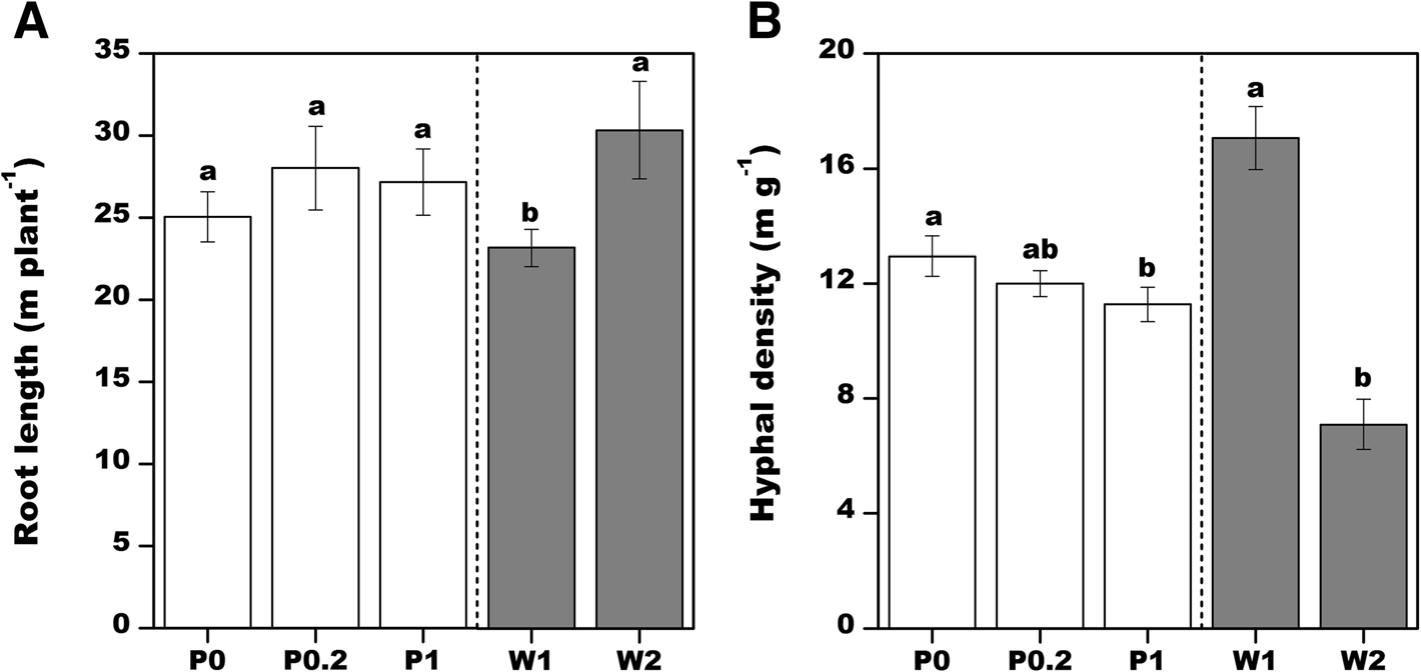
Effects of different soil phosphorus and water contents on cotton root length and hyphal density (two factor analysis of variance). Different letters above bars indicate significant differences in the phosphorus (white column) or water (gray column) contents at the 0.05 level. Error bars represent the standard error of the mean (*n* = 6) (same as in Fig. 3)

Two-way analysis of variance (Fig. 2) revealed that fertilizing with extremely high or low P concentrations was not conducive to cotton root elongation, with maximum root lengths (28 m plant^− 1^) obtained under the P0.2 treatment. Furthermore, hyphal density increased with decreasing P content, with an average hyphal density of 12.9 mg^− 1^ following the P0 treatment. In contrast, the hyphal densities after the P0.2 and P1 treatments were 7 and 12.3% lower at 12 and 11.3 m g^− 1^, respectively.

Soil water had a greater effect on cotton root and hyphal growth than P content. For example,, cotton root length and hyphal density were 23.2 m plant^− 1^ and 7.1 mg^− 1^ under W2 conditions, while they were 30.3 m plant^− 1^ and 17.06 m g^− 1^ under W1 conditions, respectively (i.e., increased by 30.6% and 2.4 times).

**Fig. 3:**
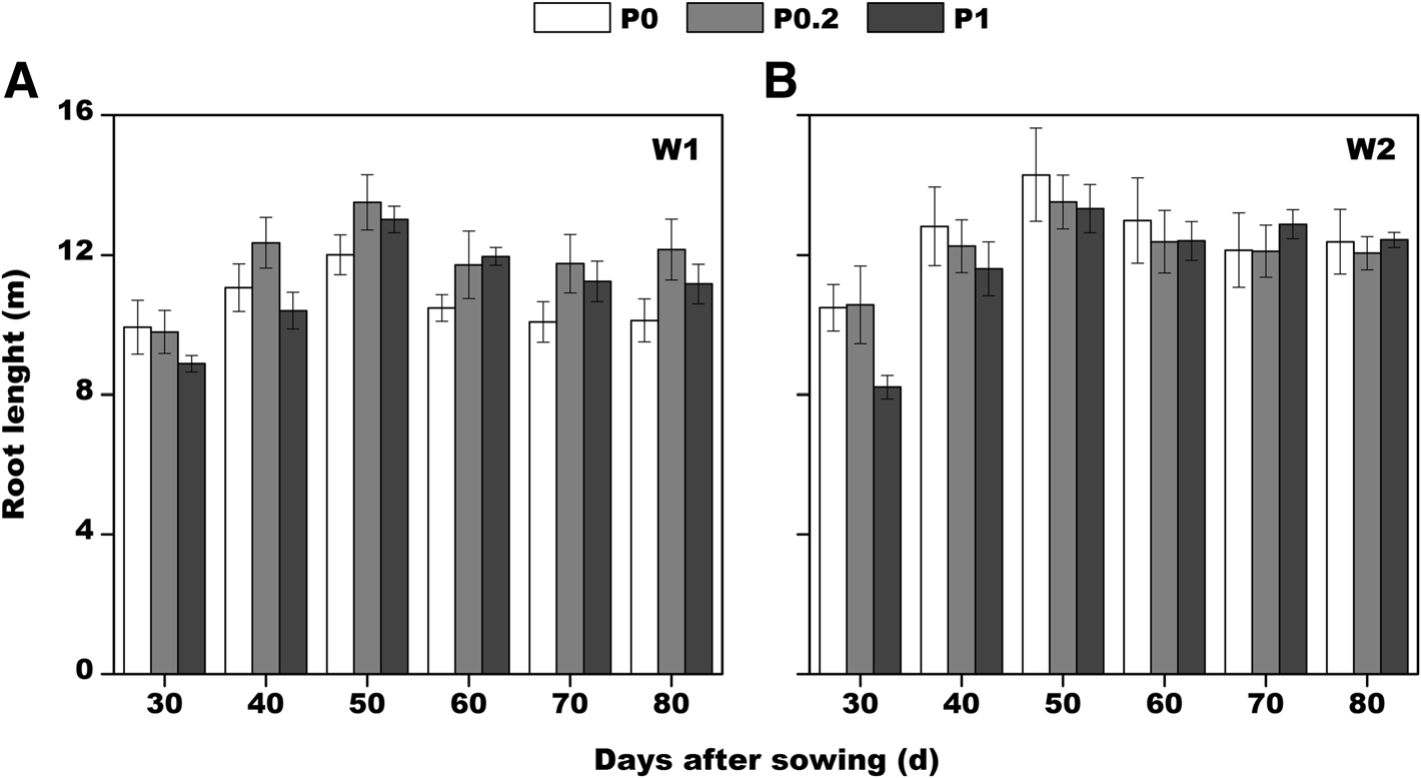
Changes in cotton root lengths over time under different treatment conditions (root mapping results)

The root length differences at 30 days after sowing were mainly due to the different P fertilizers applied during sowing, with high P concentrations inhibiting root elongation (Fig. 3). Cotton root lengths induced by P fertilizer applications were dependent on water content over time, and the P0.2 treatment promoted root elongation under W1 conditions starting from 40 days after sowing. Under W2 conditions, the influence of different P fertilizers on cotton root lengths exhibited a gradually decreasing trend as the water-treatment time increased, with almost no differences at the end of the study period (80 days after sowing).

**Fig. 4:**
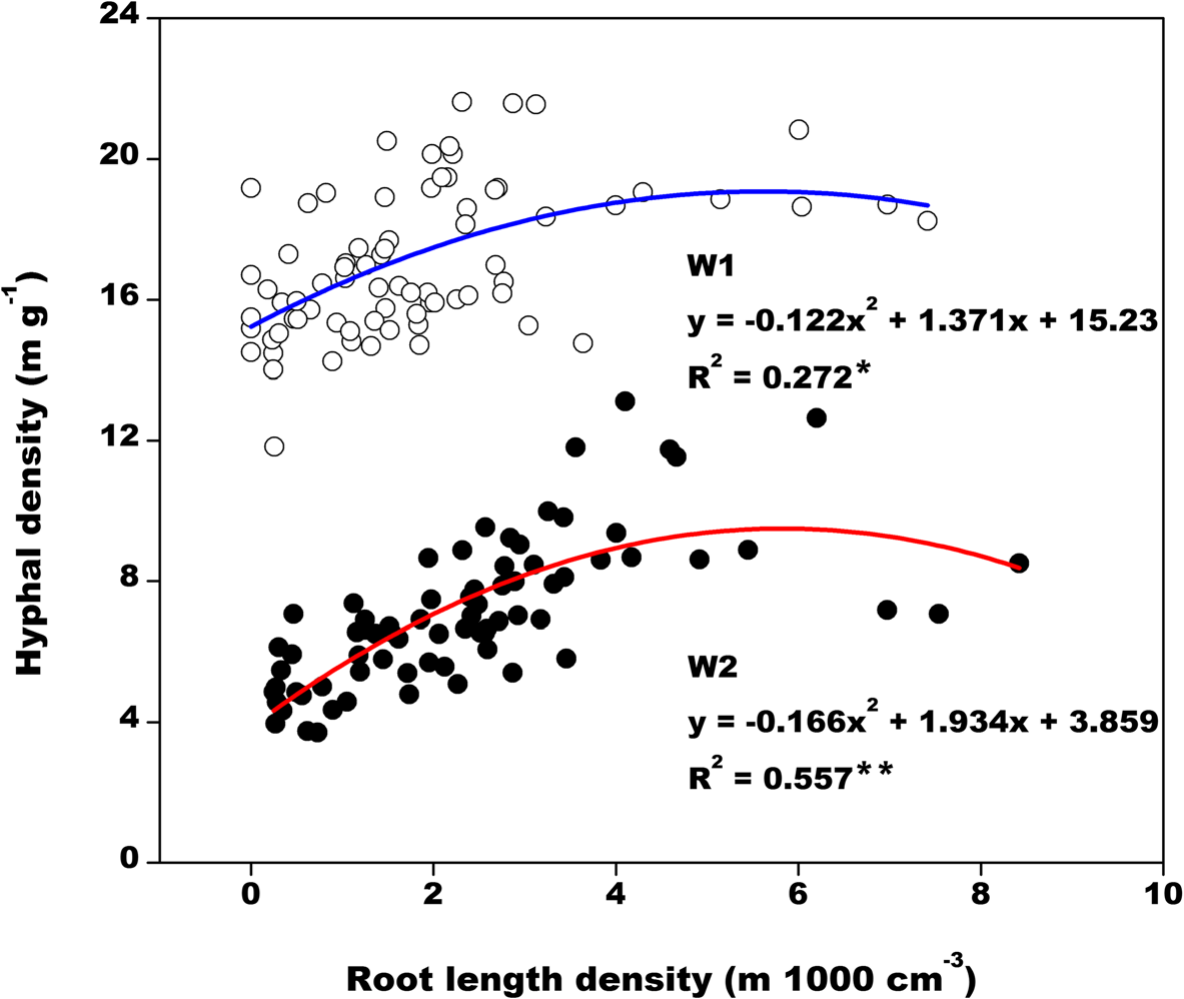
Correlation between root length density and hyphal density under different soil water conditions. ** and * indicate a significant difference at the 0.01 and 0.05 levels, respectively (*n* = 75; *P*_0.05_ = 0.226 and *P*_0.01_ = 0.294)

A synergistic relationship was observed between root length density and hyphal density (Fig. 4). The root length density was 0–5 m 1000 cm^− 3^. Meanwhile, the hyphal density increased with increasing root length density, and then tended to stabilize before finally declining at root length densities > 5 m 1000 cm^− 3^. Considering hyphal growth depends on the photosynthetic products supplied by the cotton plants, root growth is a critical factor affecting the growth of AMF associated with cotton.

### Cotton growth and phosphorus uptake

Cotton root growth was relatively high under P-deficient and/or water-limited conditions (Table 1). However, shoots grew best in response to the P0.2 and well-watered conditions. Similar results were observed for P uptake.

**Table 1:** Effects of soil water and phosphorus contents on the growth and phosphorus uptake of cotton plants

Treatment	Dry matter weight (g plant^− 1^)					P uptake (mg plant^− 1^)				
	Root	Stem	Leaf	Shoot	Total	Root	Stem	Leaf	Shoot	Total
P0										
W1	1.7bc	2.5bc	4.5c	7.0a	8.7b	5.6ab	7.8ab	15.7b	23.4bc	29.0b
W2	2.3a	1.6c	4.0c	5.6b	7.9b	8.0a	5.6b	14.8b	20.3c	28.3b
P0.2										
W1	1.3c	2.6ab	9.1a	11.7a	13.0a	4.2b	8.8ab	32.4a	41.1a	45.4a
W2	1.6bc	3.5a	6.3b	9.8a	11.4a	5.2b	10.9a	22.1ab	33.0ab	38.3ab
P1										
W1	1.3c	2.7ab	7.2b	10.0a	11.3a	5.7ab	9.9a	31.6a	41.6a	47.3a
W2	2.0ab	2.3bc	4.5c	6.8b	8.8b	8.1a	9.8a	21.7ab	31.5abc	39.7ab

Differences among six treatments were analyzed by 2 (Water) × 3 (P) ANOVA. Different letters within the same column indicate significant differences at the 0.05 level. “Shoot” indicates the cotton dry matter weight or P uptake of the stem plus leaf, and “Total” indicates the root plus shoot

**Fig. 5:**
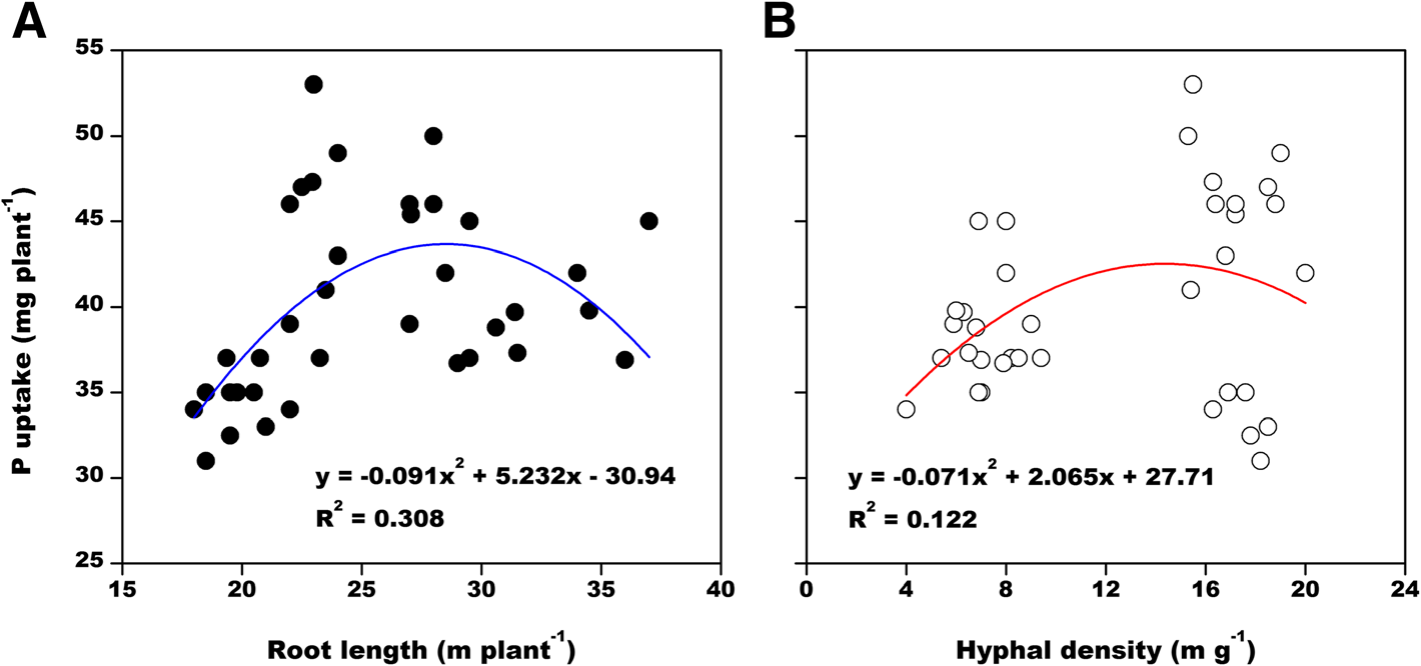
Correlation between root length (**a**) or hyphal density (**b**) and uptake of phosphorus by cotton plants (*n* = 36)

A correlation analysis indicated that a root length of 28 m plant^− 1^ and a hyphal density of 14 m g^− 1^ were critical values for the uptake of P by cotton plants (Fig. 5). At lower values, P uptake increased with increasing root length or hyphal density, whereas higher values were associated with inhibited P uptake. Although the correlations were not significant, root length had a greater effect on P uptake (R^2^ = 0.306) than hyphal density (R^2^ = 0.122).

### Regulation of soil water and phosphorus contents

**Fig. 6:**
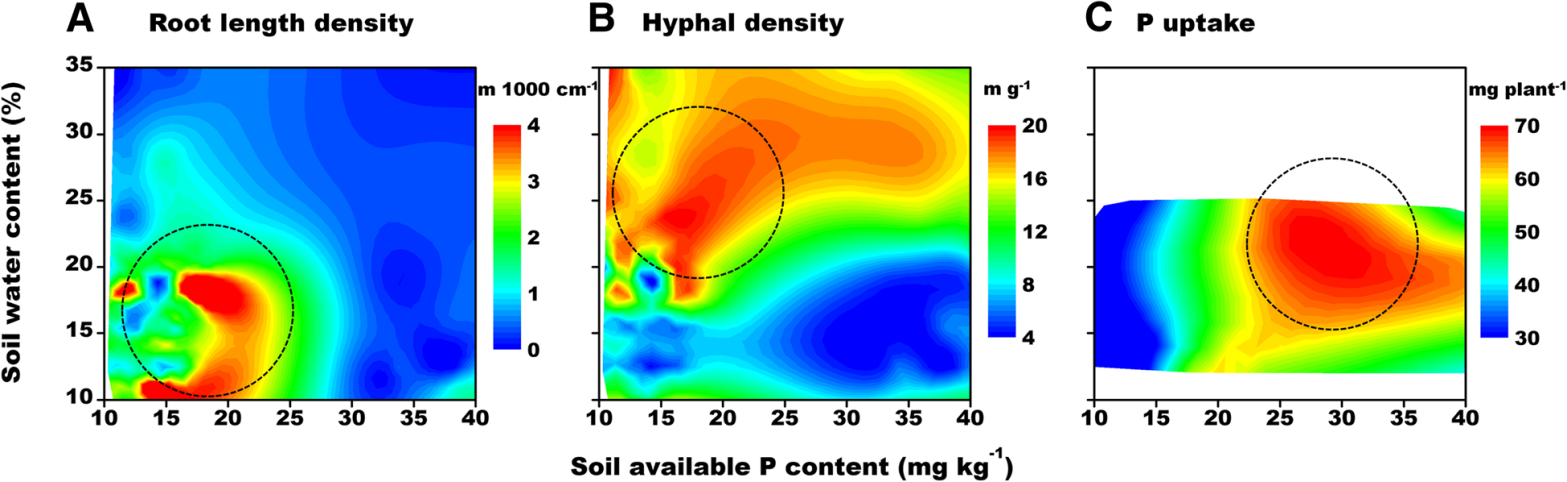
Combined effects of water and phosphorus on cotton root length density (**a**), hyphal density (**b**), and P uptake (**c**). In **c**, the soil water content is the average value in the root boxes, so the soil water content range is smaller. Areas in which the maximum values are located are indicated by a dotted circle

The relationships between soil water–P contents and root length density, hyphal density, and P uptake by cotton plants were analyzed to determine the ideal soil water and P content range that can promote cotton root and hyphal growth and simultaneously maximize P uptake (Fig. 6). Different soil water and P contents were required for maximizing root length density, hyphal density, and P uptake. The optimal soil P and water contents were 13–25 mg kg^− 1^ and < 23% for maximal cotton root elongation. In contrast, hyphal growth was highest when soil P and water contents were 12–24 mg kg^− 1^ and 20–30%, respectively. Furthermore, P uptake was optimal at soil P and water contents of 22–37 mg kg^− 1^and 18–24%, respectively.

**Fig. 7:**
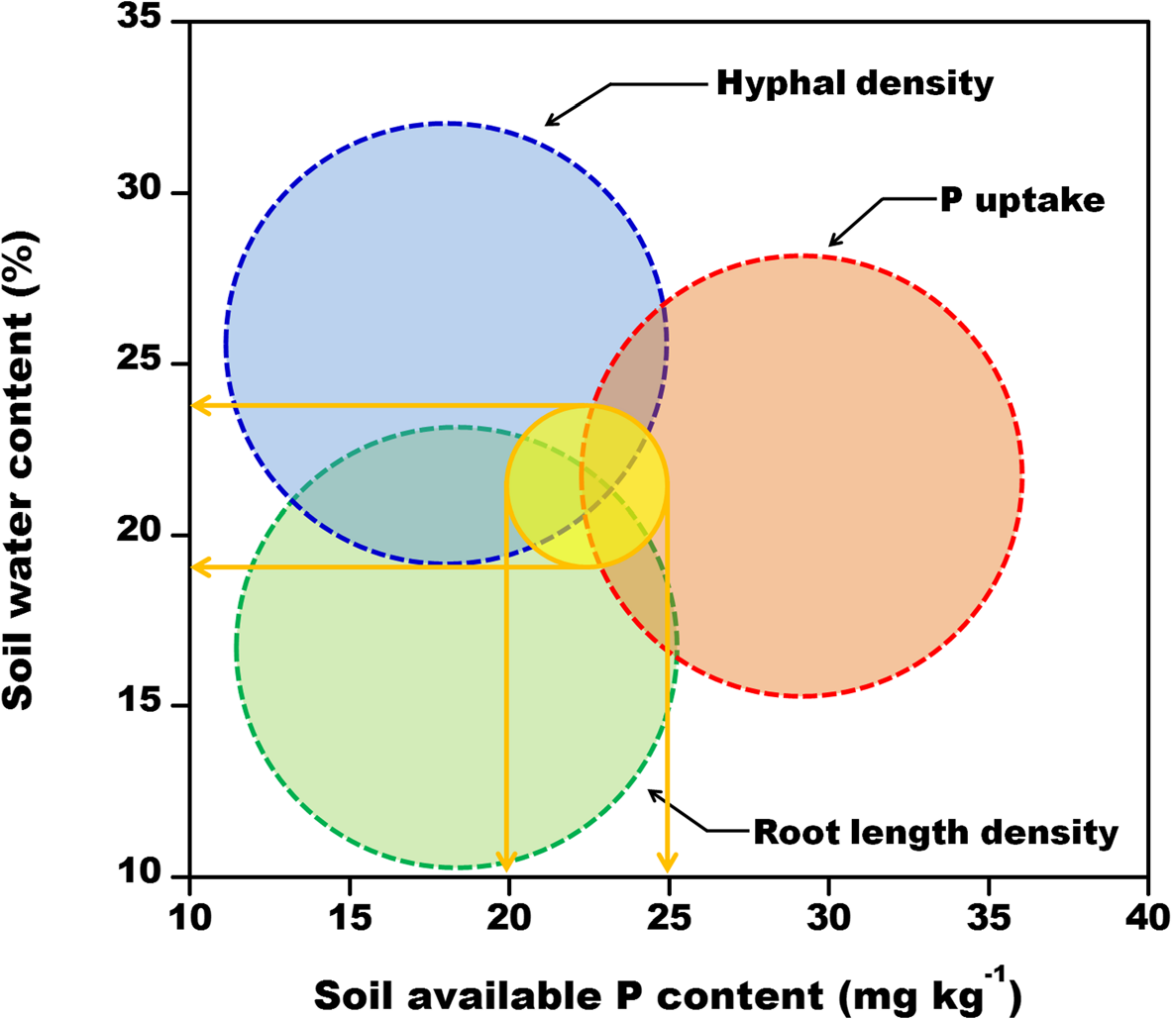
Regulation of soil water and phosphorus levels on the uptake of phosphorus by cotton plants. The positions of the different colored circles are the same as in Fig. 6, and correspond to the regions with the highest values for hyphal density (blue), P uptake (red), and root length density (green) under different soil water–phosphorus conditions. The yellow circle represents the overlapping area of the three larger circles, and corresponds to the conditions for simultaneously maximizing root length, hyphal growth, and P uptake

An analysis of the combined effects of soil water and P concentrations on root length density, hyphal density, and P uptake (Fig. 7) revealed the optimal soil P and water contents for simultaneously maximizing these three main indicators were 20–25 mg kg^− 1^ and 19–24%, respectively.

## Discussion

### Soil water had more significant effects on root and hyphal growth than phosphorus levels

Drought stress increased cotton root length (Fig. 2a), while the effects of P on cotton root length depended on the soil water condition (P0.2 promoted root elongation in well-watered soil, while the application of P had almost no influence on root length under drought conditions). Regarding the effect of P fertilizer on mycorrhizal growth, a common view is that mycorrhizal colonization and growth is inhibited with increasing P levels [16]. We also observed that hyphal density gradually decreased with increasing P concentrations. However, the soil water condition affected hyphal growth more than the applied P, with a relatively high hyphal density in well-watered soil. The water content decreased at increasing soil depths under two water treatments, and the water content in the soil profile is obviously higher under well-watered conditions than that in water-limited conditions (Additional file 1: Figure S1) also prove this result. Several studies have confirmed that soil water conditions considerably influence plant and mycorrhizal growth. For example, Ryan and Ash [30] compared wheat growth in a field under normal conditions with wheat growth in the subsequent very dry year in southern New South Wales, Australia. They observed that mycorrhizal colonization decreased from 40 to 70% to 5–16% during the dry year. Moreover, the colonization of field-grown wheat [30] and pot-grown maize [27] by AMF was low under severe drought conditions. The results of another study indicated that substantial AMF colonization can occur under well-watered and mild to moderate drought conditions [31], but it is important to note that in these experiments, plants were not exposed to extreme drought stress. Thus, our data imply that prolonged severe drought conditions seriously inhibit mycorrhizal growth. Consequently, ensuring the availability of an adequate water supply is a prerequisite for P fertilizer-regulated cotton root and mycorrhizal growth.

Soil water levels had the opposite effects on total cotton root length and average soil hyphal density, with well-watered soil decreasing root length, but increasing hyphal density. Moreover, root length and average hyphal density did not exhibit the same trends in response to P fertilizers (Fig. 2). However, in a certain root length density range (< 5 m 1000 cm^− 3^), the hyphal density increased with increasing root length density (Fig. 4; potentially under well-watered conditions). Additionally, hyphal density will exhibit a decreasing trend only when the root length density increases further (probably under drought conditions). Therefore, regarding the whole soil profile, there exists a suitable water–P range that simultaneously promotes the growth of cotton roots and hyphae. For example, for cotton plants exposed to P0.2 and W1, root length and average hyphal density were relatively high following all treatments (Fig. 2).

### Optimizing phosphorus inputs and soil water can increase cotton growth and phosphorus uptake by maximizing the efficiency of phosphorus acquisition by roots/mycorrhizae

Despite the fact drought stress promoted cotton root elongation (Fig. 2), which is theoretically conducive to the absorption of P, the cotton shoot P content was lower under drought conditions than under well-watered conditions (Table 1). Furthermore, the shoot P level was highest in plants exposed to W1 and P0.2 (Table 1). The correlation analysis revealed that cotton P uptake increased as the roots lengthened to about 28 m plant^− 1^ (Fig. 5). Longer root lengths resulted in decreased P uptake. This observation is consistent with the changes in cotton root length induced by P fertilizer under well-watered, but not drought, conditions. Therefore, P fertilizer-induced changes to roots that increase the absorption of P occurs only in well-watered soil. In drought-stressed cotton plants, the first adaptive response involves transferring photosynthates from the shoot to the roots, resulting in increased root growth, which enhances the ability of plants to absorb water. A consequence of these changes is that shoot growth is inhibited (Table 1).

Well-watered soil promotes hyphal growth and increases P uptake by cotton plants. However, increasing the P concentration of fertilizers suppresses hyphal growth, while increasing P uptake. Although hyphal density and cotton P uptake are not correlated (Fig. 5b), hyphal growth is beneficial for cotton P levels. Earlier studies confirmed that AMF increase P uptake by cowpea and capsicum only under drought and P-deficient conditions [31, 32]. Similarly, in numerous other species, P levels are enhanced in AMF-colonized plants under drought conditions, which many authors have suggested is responsible for increasing drought resistance [25, 26, 33–35]. However, it is important to note that these experiments involved only mild–moderate drought stress. In contrast, in the present study, plants were exposed to more severe drought conditions over a longer period, which inhibited the growth of mycorrhizal fungi and decreased the contribution of the AMF P-uptake pathway. We did not use moderate or mild drought treatments. Our justification for this is that in contrast to the aforementioned studies in which increased P uptake via AMF colonization improved drought resistance, the uptake of P after exposing perennial ryegrass and wheat to drought stress is reportedly unaffected by the presence of AMF [28, 29]. Similarly, inoculations with AMF did not affect maize growth under drought conditions [27].

Although there is some inconsistency in hyphal and root growth responses to water–P contents, it is still important to consider the overall effects of different water and P levels on root elongation, hyphal growth, P uptake, cotton growth, and even nutrient input costs. A combination of W1 and P0.2 conditions maximizes root and mycorrhizal development, thereby ensuring improved cotton growth and increased P uptake. Under the conditions tested during this study, the ideal soil water and available P contents were19–24% and 20–25 mg kg^− 1^, respectively (Fig. 7). To further quantify the effects of different water and P conditions on AMF and P uptake by cotton roots, it is very important that the contributions of the direct root P-uptake and AMF P-uptake pathways following different water and P treatments are determined.

## Conclusions

Drought stress inhibited hyphal growth compared with well-watered condition. Additionally, P fertilizer-regulated cotton root elongation occurred only under well-watered conditions. Too much or too little P fertilizer inhibited cotton root elongation. In contrast, root elongation was essentially unaffected by P fertilizers under drought conditions. The effects of P on hyphal growth exhibited a similar trend regardless of soil water conditions (i.e., hyphal density decreased as P content increased). Under well-watered conditions, the application of P fertilizers significantly increased cotton P uptake, but there was no significant difference between the P0.2 and P1 treatments. Therefore, there is some inconsistency in root and hyphal growth as well as cotton P uptake in response to soil water–P changes. Therefore, the soil water–P contents (i.e., soil water and P contents of 19–24% and 20–25 mg kg^− 1^, respectively) should be controlled to simultaneously maximize cotton root/mycorrhizal growth and P uptake by cotton plants.

## Methods

### Biological materials and soil

Seeds of *Gossypium hirsutum*cv. XLZ50, which is currently the major cultivated cotton genotype in Xinjiang, were obtained from the Xinjiang Academy of Agricultural Sciences, China. Cotton plants were grown in a gray desert soil collected from the Xiaoguai Experimental Station of the Xinjiang Institute of Ecology and Geography, Chinese Academy of Sciences in Urumqi, China. The soil collected from a field that had not been used to grow crops was air-dried and then filtered through a 2-mm sieve. An analysis prior to sowing revealed the soil chemical properties were as follows: 16.7 mg kg^− 1^extracted mineral nitrogen, pH (H_2_O) 8.1, 1.33 g cm^−3^soil density, 8.2 mgkg^− 1^ Olsen-P, 208.9 mg kg^− 1^ NH_4_OAc-extracted potassium, and 5.3 g kg^− 1^ organic matter.

### Experimental design

This study was conducted in a greenhouse over 90 days from June to September in 2015 and 2016. Experiments comprised two water contents and three P contents in a 2 × 3 factorial design. The two water contents were 80% of field water capacity (W1, well-watered) and 40% of field water capacity (W2, drought). The three P contents were 0 g P_2_O_5_ kg^− 1^ (P-deficient, P0), 0.2 g P_2_O_5_ kg^− 1^ (middling P, P0.2), and 1 g P_2_O_5_ kg^− 1^ (excess P, P1). Three replicates were analyzed for each of the six treatment combinations.

**Fig. 8:**
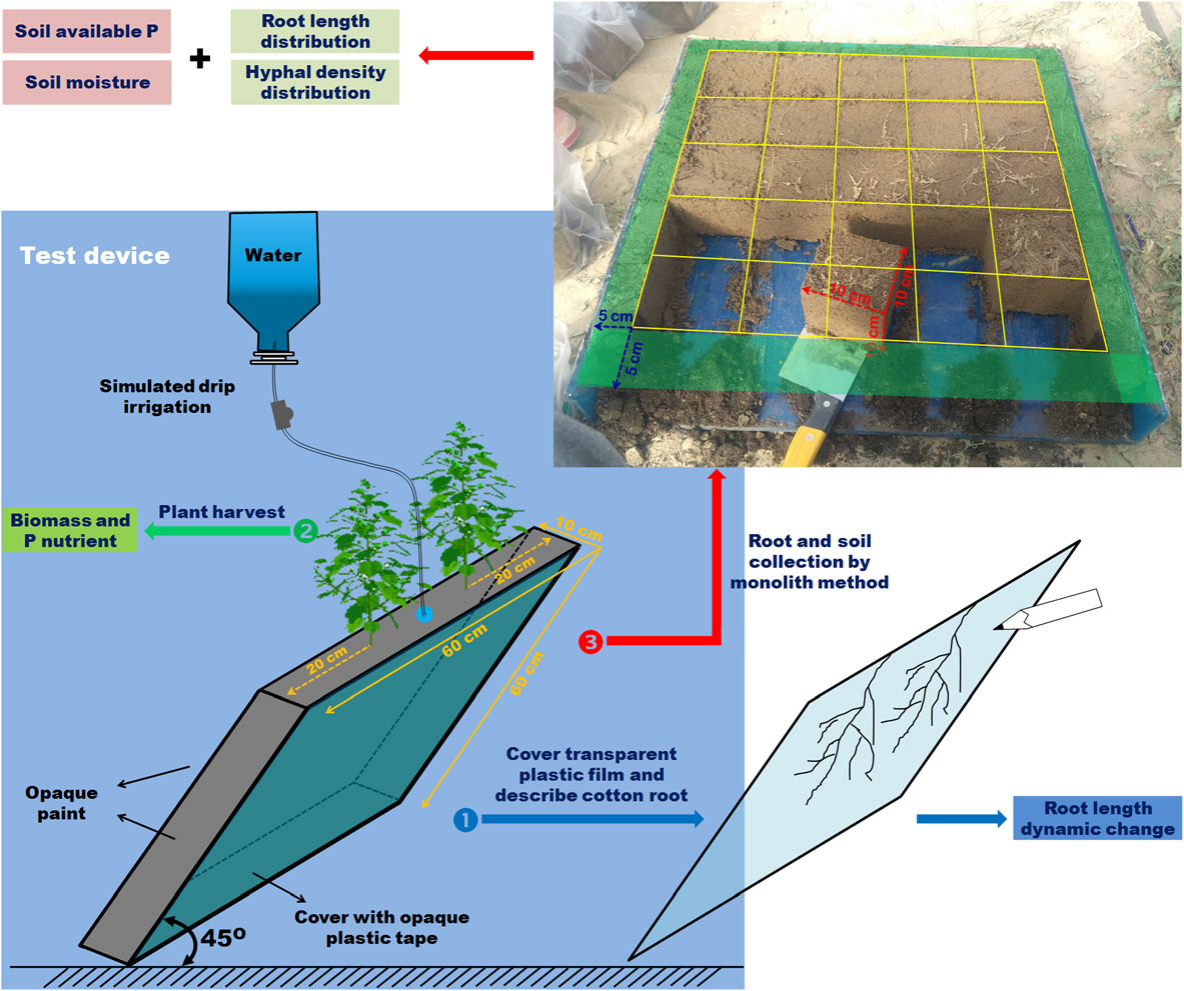
Root box specifications and diagram of the cotton cultivation method. Root boxes were made of 8-mm thick glass (length and width: 60 cm; internal thickness: 10 cm), with an opening on top for planting cotton. Four sides of each root box were covered with opaque paint, and the remaining side (60 cm × 60 cm) was covered with opaque plastic, which was removed to observe the root morphology. The root boxes were maintained at a 45° angle between this side and the ground to ensure the cotton roots will grow close to the glass wall. Each root box consisted of two cotton plants separated by 20 cm. A drip irrigation system was simulated to accurately control the flow of water. During the experiment, the opaque plastic was removed and replaced with a transparent plastic film, after which the cotton root architecture was traced using a black marker and then scanned to quantify the root length changes (➊). At the end of the experiment, the shoots were harvested, additionally, to examine cotton growth and Puptake (➋). The soil in each root box was cut into cubes with 10-cm sides (25 blocks per root box). The roots were collected after the soil samples were passed through a sieve for root length measurements, while the soil samples were collected for determining hyphal density, soil water level, and available P content (➌)

Soil (38 kg) was weighed in a plastic bag and then thoroughly mixed with KH_2_PO_4_ (0, 0.2, or 1 g P_2_O_5_ kg^− 1^) and urea (0.25 kg N kg^− 1^) before being added to glass root boxes (width and height: 60 cm; thickness: 10 cm) (Fig. 8). The root boxes were divided into three groups with each being filled with 0.2 g P_2_O_5_ kg^− 1^ P fertilizer, 1 g P_2_O_5_ kg^− 1^ P fertilizer, or no P fertilizer.

Cotton seeds were disinfected with 10% (*v*/v) H_2_O_2_ for 10 min and 70% (v/v) ethanol for 3 min and then rinsed eight times with sterile deionized water. After a 2-day imbibition in water at 27 °C in darkness, six pre-germinated seeds were sown in pots. The plants were thinned to two seedlings per pot after 10 days. Soil water was maintained at 80% of field water capacity as determined gravimetrically by weighing the pots every 3 days and adding water as necessary with a simulated drip irrigation system. Water treatments were initiated 30 days after sowing, with root boxes for each P fertilizer treatment divided into two groups. For the remainder of the experiment, the soil water level of half of the root boxes was kept at 80% of field water capacity, while the soil water content of the other half was lowered to 40% of field water capacity.

### Sample harvest and analysis

The root systems were analyzed when initiating the water treatments and then re-analyzed every 10 days for 60 days (six times in total) (Fig. 8➊). The roots were then scanned with a digital scanner (Epson V700, Djakarta, Indonesia) at 200 dpi with grayscale pixels. The resulting images were saved as TIF files and then analyzed using the DELTA-T SCAN program (version 1.0) (Delta-T Devices, Burwell, UK).

At the end of the 60-day water treatments, the shoots were cut and divided into leaves and stems (Fig. 8➋). All samples were heated at 105 °C for 30 min and then dried at 70 °C until a constant weight was attained. The dry weight was recorded and subsamples were used to measure the P content according to the standard vanado-molybdate method [36].

After harvesting the shoots, the roots were collected using a published monolith method [37] (Fig. 8➌). Soil cubes with 10 cm sides (1000 cm^3^) were cut individually in a soil volume of 50 cm × 50 cm × 10 cm. The 25 monoliths prepared for each root box were sieved through a stainless steel mesh (1 mm diameter) and the roots were rinsed with water. The collected samples were then stored at − 20 °C until the root lengths were measured.

Soil samples were collected from each soil block after the roots were sieved, with some being used to measure soil water content according to a drying method, while others were air-dried and passed through a sieve (1 mm diameter) and analyzed. The available P was extracted from soil using 0.5 M NaHCO_3_ (2.5 g soil in a 50-ml solution shaken at 25 °C for 30 min) and the inorganic P was colorimetrically measured using an established molybdate–ascorbic acid method [38]. The hyphal density in the soil was measured using a modified membrane filter technique [39].

Roots collected from each soil block were also analyzed with a digital scanner. Root samples were placed in a glass rectangular dish (200 mm × 150 mm) containing a 4–5-mm layer of water to untangle the roots and minimize root overlap. When necessary, the roots of one soil block were separated into subsamples until they could be placed in the dish. The images were analyzed using the DELTA-T SCAN program. The root fractions were subsequently combined and dried at 70 °C until a constant weight was attained. Sample weight and P content were then recorded.

### Data analysis

Data underwent a 2-way analysis of variance (SAS 8.0 software, SAS Institute, 1998). Means in the different treatments were compared based on the least significant difference at the 0.05 level of significance. The spatial distributions of cotton root length density and hyphal density in the soil profiles are presented as wireframe diagrams (Surfer 9.0 software). The mean root length per plant (m plant^− 1^) was calculated by dividing the total root length for the 25 soil blocks by 2 (i.e., the number of sampled plants).

## Additional file

Additional file 1: **Figure S1.** Soil water content under different water treatment. (TIF 692 kb)

## Abbreviations

AMF: Arbuscular mycorrhizal fungi
DAS: Days after sowing
