# Supplementary figures and images for: Simultaneously maximizing root/mycorrhizal growth and phosphorus uptake by cotton plants by optimizing water and phosphorus management
Source: BMC Plant Biol. 2018 Dec 5;18:334. doi: 10.1186/s12870-018-1550-8 (PMC6280356; doi:10.1186/s12870-018-1550-8)

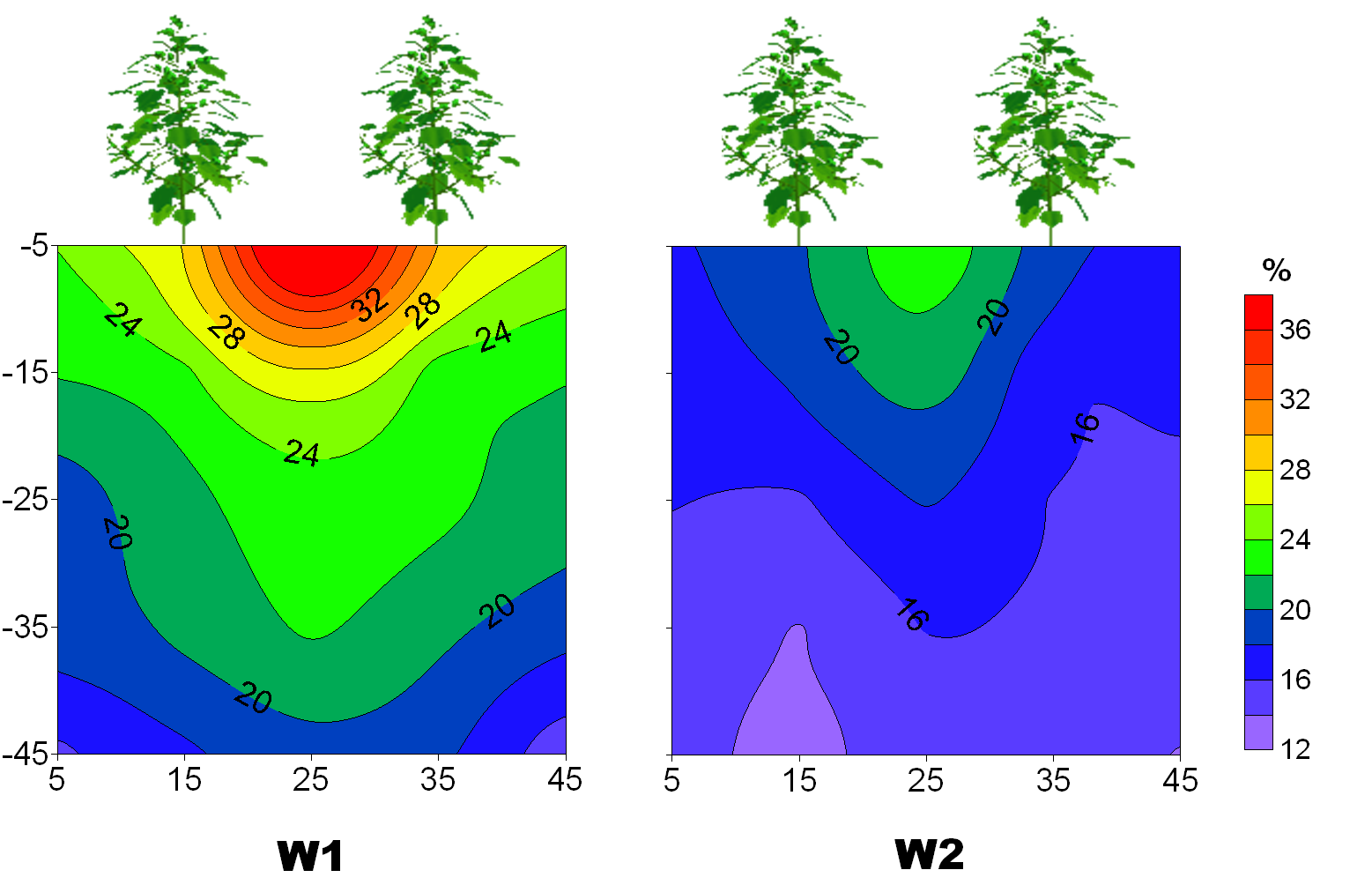

Supplement: Supplementary file 1 — Figure S1. Soil water content under different water treatment. (TIF 692 kb) [file 12870_2018_1550_MOESM1_ESM.tif]
